# An integrative evaluation of circadian gene TIMELESS as a pan-cancer immunological and predictive biomarker

**DOI:** 10.1186/s40001-023-01519-3

**Published:** 2023-12-05

**Authors:** Yaocheng Yang, Xianzhe Tang, Zhengjun Lin, Tao Zheng, Sheng Zhang, Tang Liu, Xiaolun Yang

**Affiliations:** 1 Department of Orthopedics, The Second Xiangya Hospital, Central South University, 136 Renmin Middle Road, Changsha, Hunan 410011 People’s Republic of China; 2 Department of Orthopedics, Chenzhou First People’s Hospital, Chenzhou, Hunan China; 3 Department of Oral and Maxillofacial Surgery, The Second Xiangya Hospital, Central South University, Changsha, Hunan China; 4 Department of Stomatology, The Second Xiangya Hospital, Central South University, 136 Renmin Middle Road, Changsha, Hunan 410011 People’s Republic of China

**Keywords:** TIMELESS, Pan-cancer, Circadian rhythm, Prognosis, Immunological biomarker

## Abstract

**Background:**

The gene TIMELESS, which is involved in the circadian clock and the cell cycle, has recently been linked to various human cancers. Nevertheless, the association between TIMELESS expression and the prognosis of individuals afflicted with pan-cancer remains largely unknown.

**Objectives:**

The present study aims to exhaustively scrutinize the expression patterns, functional attributes, prognostic implications, and immunological contributions of TIMELESS across diverse types of human cancer.

**Methods:**

The expression of TIMELESS in normal and malignant tissues was examined, as well as their clinicopathologic and survival data. The characteristics of genetic alteration and molecular subtypes of cancers were also investigated. In addition, the relationship of TIMELESS with immune infiltration, tumor mutation burden (TMB), microsatellite instability (MSI), and drug sensitivity was illustrated. Immunohistochemistry (IHC) was used to validate the expression of TIMELESS in clinical patients with several types of cancer.

**Results:**

In contrast to the matching normal controls, most tumor types were found to often overexpress TIMELESS. Abnormal expression of TIMELESS was significantly related to more advanced tumor stage and poorer prognosis of breast cancer, as well as infiltrating immune cells such as cancer-associated fibroblast infiltration in various tumors. Multiple cancer types exhibited abnormal expression of TIMELESS, which was also highly correlated with MSI and TMB. More crucially, TIMELESS showed promise in predicting the effectiveness of immunotherapy and medication sensitivity in cancer therapy. Moreover, cell cycle, DNA replication, circadian rhythm, and mismatch repair were involved in the functional mechanisms of TIMELESS on carcinogenesis. Furthermore, immunohistochemical results manifested that the TIMELESS expression was abnormal in some cancers.

**Conclusions:**

This study provides new insights into the link between the circadian gene TIMELESS and the development of various malignant tumors. The findings suggest that TIMELESS could be a prospective prognostic and immunological biomarker for pan-cancer.

**Supplementary Information:**

The online version contains supplementary material available at 10.1186/s40001-023-01519-3.

## Introduction

Cancer represents one of the most substantial hazards to human existence and stands as a global public health predicament [1]. The Global Burden of Disease Study reports that in 2019, there were 10 million cancer-related deaths and 23.6 million new cancer cases worldwide [2]. Consequently, it is utmost importance to perform a comprehensive analysis of pan-cancer expression for certain selected genes and determine how their expression relates to clinical traits, survival chances, and putative molecular mechanisms. Fortunately, there now exists a basis for doing pan-cancer analysis thanks to vast-scale and multi-omics cancer data sets, such as TCGA and GEO, which provide functional genomics data sets of numerous types of tumors [3, 4].

The circadian clock protein TIMELESS was first discovered in the Drosophila melanogaster [5, 6]. Soon after, in mammals, TIMELESS also plays a variety of roles in physiological and pathological processes, including DNA replication, DNA damage response, cell cycle, and circadian rhythm [7–9]. A universal intrinsic timekeeping system is the circadian rhythm [10]. The relationship between circadian genes—including ARNTL, CLOCK, and PER1/2/3—and the onset of cancer has been thoroughly studied over the past few decades [11–13]. Nowadays, night shift employment has been categorized by the International Agency for Research on Cancer (IARC) Monographs Vol 124 Working Group as a potential human carcinogen (Group 2A) in June 2019 [14]. However, shift work with circadian disruption has been considered as a vital carcinogenic risk factor for various cancer [15–18]. Similar to TIMELESS, an important component of the circadian clock, these cancers include non-small cell lung cancer [19], colorectal cancer [20], nasopharyngeal carcinoma (NPC) [21], gliomas [22], and breast cancer [23], all of which display abnormal expression of TIMELESS and exhibit increased tumor aggressiveness. Although there have been an increasing number of research linking TIMELESS to human illnesses, notably malignant tumors, there is currently no pan-cancer analysis about the role of TIMELESS in the management of cancer. Consequently, the objective of this study is to thoroughly analyze bioinformatic data to investigate the function of TIMELESS in multiple cancer types.

To examine the function and underlying mechanism in tumorigenesis, we created a pan-cancer expression study of TIMELESS using a number of open-access databases. We also compared the correlations between TIMELESS expression and clinical characteristics, survival status, genetic alteration, immune cell infiltration, microsatellite instability (MSI), Tumor mutation burden (TMB), drug sensitivity, and relevant signaling pathway of malignant tumors. In addition, using immunohistochemistry (IHC), we confirmed the expression of TIMELESS in clinical patients across several tumor types. The study of TIMELESS could help in forecasting the survival and benefits of immunotherapeutic in cancer patients, according to our data, suggesting that TIMELESS plays a critical role in the development of various human cancers.

## Materials and methods

### Gene and protein expression analysis of TIMELESS

First, we used the Human Protein Atlas (HPA) database (version 20.1) to create a TIMELESS mRNA expression plot. Next, with the Tumor Immune Estimation Resource 2.0 (TIMER2.0) online database, we scanned the expression difference of TIMELESS between malignant tumors and corresponding normal tissues for the various cancers or specific tumor subtypes of the TCGA study. We also compared the expression of these tumor tissues to the normal tissues that matched them in the GTEx (Genotype-Tissue Expression) database (*P* value cutoff = 0.01, log_2_FC cutoff = 1), using the GEPIA2 (Gene Expression Profiling Interactive Analysis, version 2) website for certain tumors without normal tissues or with only a small number of normal tissues.

In addition, we created violin plots of the TIMELESS expression in varied clinical stages of all TCGA tumors using the GEPIA2. The box or violin plots were created using the log [TPM (transcripts per million) + 1] converted expression data. In addition, we examined the expression profiles of TIMELESS in various malignancies and paired normal cell lines using the BioGPS database to analyze [24]. Furthermore, we explored protein and phosphoprotein expression utilizing the CPTAC (Clinical Proteomic Tumor Analysis Consortium) data set of the UALCAN portal, a dynamic web tool for the evaluation of Omics data. By inserting “TIMELESS” here, we investigated the expression level of the total protein of TIMELESS in primary tumor versus normal tissues, including glioblastoma multiforme, hepatocellular carcinoma, lung adenocarcinoma (LUAD), uterine corpus endometrial carcinoma (UCEC), clear cell renal carcinoma (RCC), and pancreatic adenocarcinoma. The abbreviations of TCGA cancer were shown in Abbreviations list.

### Survival prognosis analysis

Then, statistics on TIMELESS' overall survival (OS) and disease-free survival (DFS) significance maps across all TCGA cancers were obtained using GEPIA2. To separate the cohorts with high and low expression, the cutoff-high and cutoff-low values (50%) were employed as the expression cutoffs. With GEPIA2, the log-rank test and survival plots were likewise collected.

### Genetic alteration analysis

We visited the cBioPortal website and decided to look into the "TCGA Pan-Cancer Atlas Studies" in order to learn more about the mutation rate and genetic modification characteristics of TIMELESS. The results of the alteration frequency, mutation type, and copy number alteration (CNA) across all TCGA tumors were displayed. For the UCEC cancer patients with or without the TIMELESS genetic change, we also gather information on the variances in overall, disease-free, progression-free, and disease-free survival using the "Comparison" module. In addition, log-rank *P* values for Kaplan–Meier plots were created.

### Immune infiltration analysis

Immune cells that infiltrate tumors are important elements of the tumor microenvironment and have been directly connected to the development, growth, or spread of cancer. Through the TIMER2.0 database, the correlation between TIMELESS expression and immune infiltrates was explored across all TCGA tumors. The MCPCOUNTER, EPIC, and TIDE algorithms were used to choose the immune cells of cancer-associated fibroblasts (CAFs) for immune infiltration estimations. The purity-adjusted Spearman's rank correlation test was used to determine the *P* values and partial correlation (cor) values. Results visualizations in the form of a heatmap and scatter plot were employed. Utilizing TIMER2, the immune-infiltrating cell was evaluated. The characteristics of correlation between TIMELESS expression and cell types, immunostimulators, immunoinhibitors, major histocompatibility complex (MHC) molecules in human being, chemokines, and receptors for various cancer types were also thoroughly investigated using TISIDB (tumor-immune system interactions database).

### Relationship between TMB and MSI and the expression of TIMELESS

Because it reveals the frequency of modification in a particular cancer type, TMB has been recognized as a significant biomarker that is strongly linked to the effectiveness of cancer immunotherapy [25]. It has also been determined that MSI, a genomic instability brought on by a flaw the DNA mismatch repair (MMR) machinery, is a crucial marker of how well cancer responds to immunotherapy [26]. Sangerbox was used to carry out the correlation analysis of the TIMELESS expression with TMB and MSI using Spearman's technique. The ordinate of figure represents various cancer types, the horizontal axis displays the correlation coefficient between TIMELESS and TMB and MSI, the magnitude of the correlation coefficient is represented in the figure by the size of the dots, and the significance of the *P* value is indicated by the different colors.

### Relationship between TIMELESS expression and drug sensitivity

The CallMiner™ online database provided the NCI-60 compound activity data with RNA-seq expression profiles that we used to investigate the relationship between the expression level of TIMELESS and treatment sensitivity in pan-cancer. Using the R packages "limma", "impute", "ggplot2", and "ggpubr", the relationship between TIMELESS expression and the sensitivity of numerous chemotherapeutic medications licensed by the FDA was examined and visualized.

### TIMELESS-related gene enrichment analysis

Through the STRING database, we first obtained the experimentally determined TIMELESS-binding proteins that were accessible. Then, utilizing information from all TCGA cancers and normal tissues, GEPIA2 was used to examine the top 100 TIMELESS-correlated targeting genes. The “correlation analysis” module of GEPIA2 was also used to get a pairwise gene Pearson correlation study of TIMELESS and specific genes. In addition, the heatmap data of the chosen genes was also displayed using TIMER2. The R packages "org.Hs.eg.db," "clusterProfiler," and "enrichplot" were also used to evaluate TIMELESS-related enriched pathways and GO and KEGG functional annotations.

### Experimental validation using immunohistochemical staining of TIMELESS

For TIMELESS expression analysis in tumor tissues, three types of the human tumor with three specimens for each one, including breast cancer, lung cancer, and renal clear cell carcinoma, were obtained from the Department of Pathology, the Second Xiangya Hospital, Central South University. The immunohistochemistry staining was implemented with the TIMELESS antibody (ab109512, Abcam), according to their manufacturer’s protocols. Furthermore, we also downloaded immunohistochemical images of six kinds of tumor tissues from HPA, including breast cancer, colorectal cancer, lung cancer, liver cancer, renal cancer, and thyroid cancer.

### Statistical analysis

GraphPad Prism (Version 8.4.2 for Windows) was used to analyze the data provided from the cBioPortal regarding the correlation between the copy number alteration of TIMELESS and its level of mRNA expression. Spearman's correlation analysis was calculated between the TIMELESS expression and TMB and MSI. Statistics were considered significant for *P* values under 0.05.

## Result

### Expression of TIMELESS in human pan-cancer

**Fig. 1:**
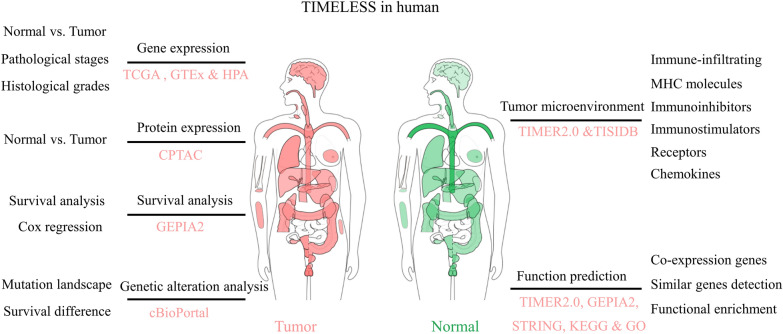
Workflow of this study and the body map of TIMELESS median expression level in human beings from GEPIA2

To examine the roles and potential mechanisms of TIMELESS in the pathogenesis or clinical survival prognosis of various types of cancer, we performed pancarcinoma analyses of TIMELESS utilizing the TCGA, GTEx, and UCSC projects, taking into account key biological processes, genetic changes, immune infiltration, as well as variations in expression levels and survival. Figure 1 depicts the study's methodology and the distribution of TIMELESS expression levels in humans from GEPIA2.

**Fig. 2:**
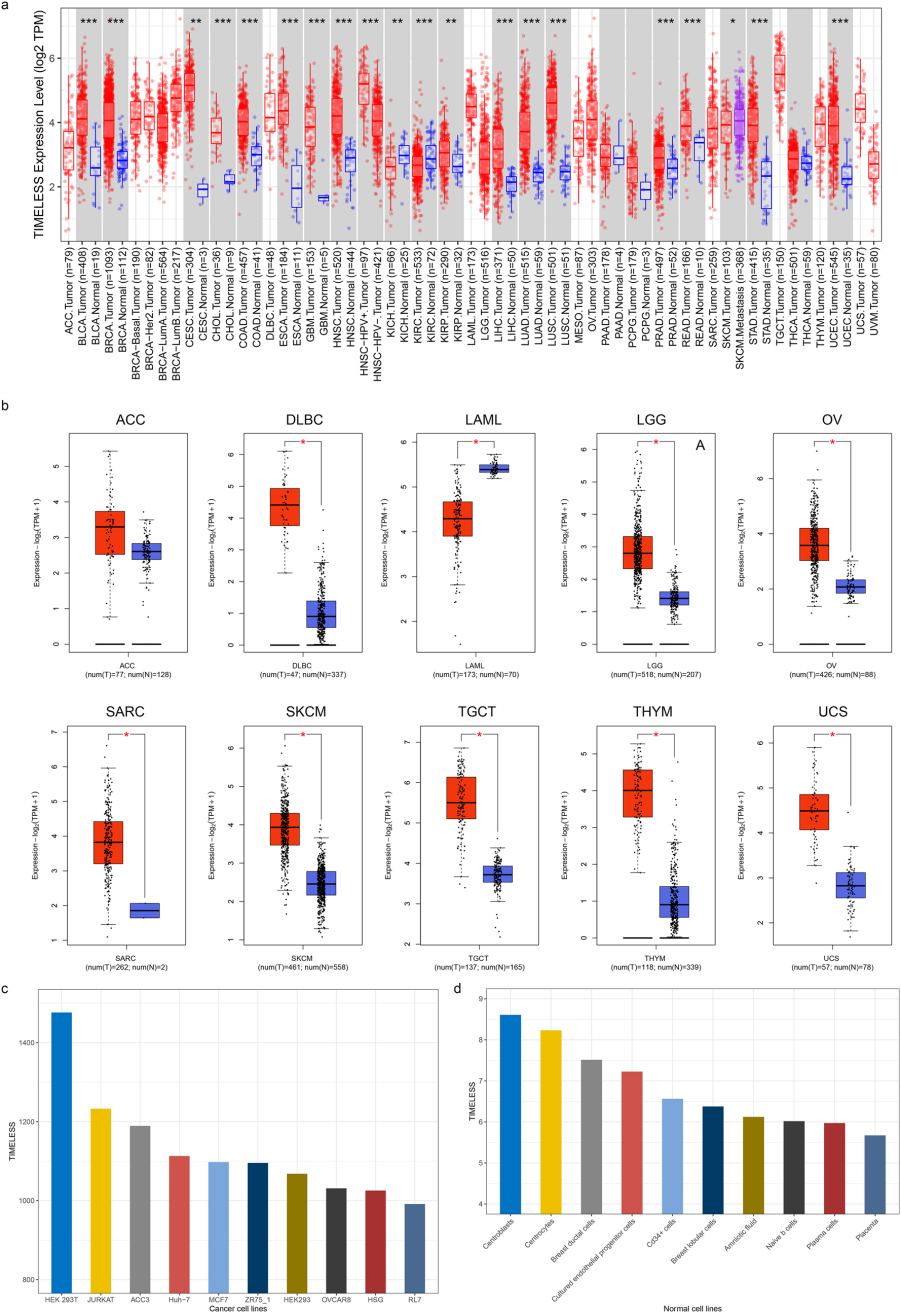

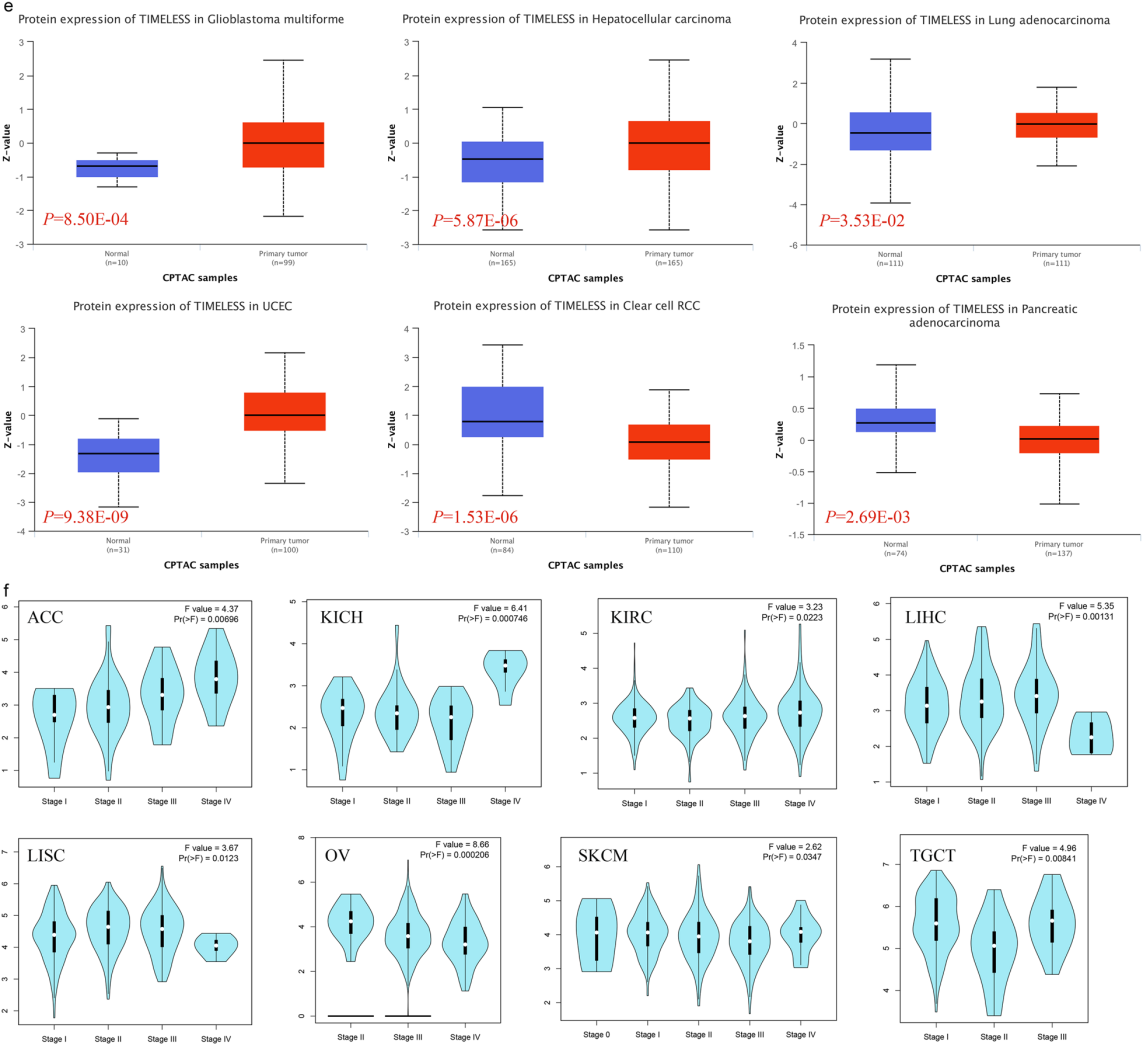
TIMELESS expression levels in human cancers. **a** TIMELESS expression levels in different cancer types from the TCGA database analyzed by the TIMER2.0 database. **b** TIMELESS expression in several cancers and paired normal tissue in the GEPIA2 database. (*P < 0.05, **P < 0.01, ***P < 0.001). **c** Expression of TIMELESS in different cancer cell lines analyzed by the BioGPS database. **d** Expression of TIMELESS in normal tissue analyzed by the BioGPS database. **e** Based on the CPTAC database, the expression level of TIMELESS total protein between normal tissue and primary tissue of glioblastoma multiforme, hepatocellular carcinoma, lung adenocarcinoma, UCEC, clear cell renal carcinoma and pancreatic adenocarcinoma were also analyzed. **f** Based on the TCGA data, the expression levels of the TIMELESS gene were analyzed by the main pathological stages (stage I, stage II, stage III, and stage IV) of ACC, KICH, KIRC, LIHC, LISC, OV, SKCM and TGCT. Log2 (TPM + 1) was applied for log-scale

The expression status of TIMELESS across diverse cancers was examined using TIMER2.0. According to the data from the TCGA and GTEx cohorts, as shown in Fig. 2a and Additional file 2: Table S1, TIMELESS expression was considerably higher in the following tissues compared to control tissues: in BLCA, BRCA, CESC, CHOL, COAD, ESCA, GBM, HNSC, KICH, KIRC, KIRP, LIHC, LUAD, LUSC, PRAD, READ, STAD, and UCEC. We further assessed the expression differential of TIMELESS between the normal and malignant tissues of ACC, DLBC, LAML, LGG, OV, SARC, SKCM, TGCT, THYM, and UCS after using the normal tissue of the GTEx data set as control (Fig. 2b and Additional file 2: Table S1, *P* < 0.01). Using the BioGPS database, we looked into the expression of TIMELESS in several cancer cell lines as well as normal tissues, and we discovered that TIMELESS was highly expressed in practically all cancer cell lines. The top ten cancer cell lines with the greatest TIMELESS expression levels, including HEK 293 T, JURKAT, ACC3, and others, are displayed in Fig. 2c. Comparing different cell types, immune cells exhibit TIMELESS to the greatest extent (Fig. 2d).

The RNA expression of TIMELESS in different tissues was also examined using the HPA database. Immunological tissues, such as bone marrow, thymus, tonsil, and lymph node have the highest levels of TIMELESS expression (Additional file 1: Fig. S1a–d). According to the CPTAC data set, TIMELESS total protein was expressed more strongly in the primary cancer tissues of glioblastoma multiforme, hepatocellular carcinoma, lung adenocarcinoma, and UCEC (Fig. 2e) compared to normal tissues, but less strongly in clear cell renal carcinoma and pancreatic adenocarcinoma (Fig. 2e). In addition, the expression of TIMELESS phosphoprotein has significantly risen in the initial cancer tissues of clear cell renal carcinoma, lung adenocarcinoma, hepatocellular carcinoma, and so on (Additional file 1: Fig. S1e). Furthermore, the pathological stage plot module of GEPIA2, which includes ACC, KICH, KIRC, LIHC, LISC, OV, SKCM, and TGCT, revealed a link between TIMELESS expression and the pathological stages of malignancies (Fig. 2f,  P < 0.05).

### Survival analysis

**Fig. 3:**
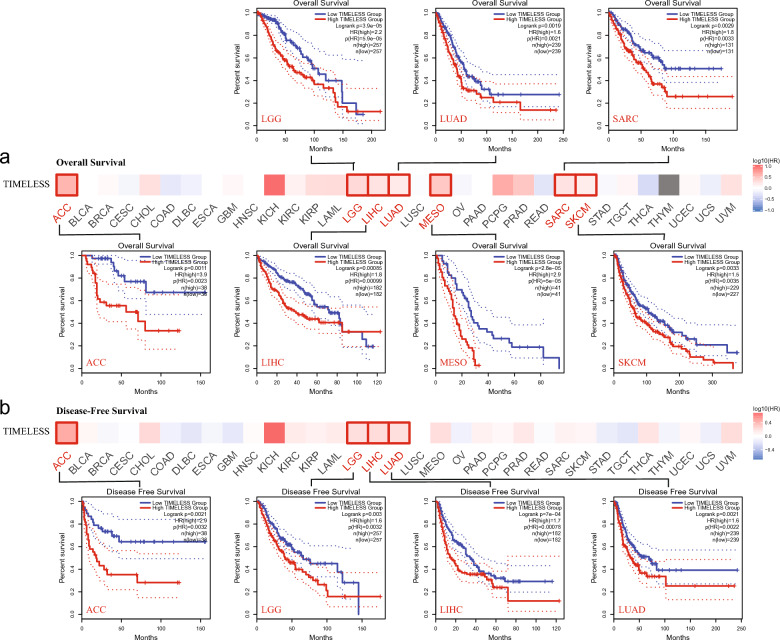
Survival analysis of TIMELESS in pan-cancers. Correlation between TIMELESS gene expression and survival prognosis of cancers in TCGA. We used the GEPIA2 tool to perform overall survival (**a**) and disease-free survival (**b**) analyses of different tumors in TCGA by TIMELESS gene expression. The survival map and Kaplan–Meier curves with positive results are given

By classifying the cancer cases into high-expression and low-expression cohorts based on the expression levels of TIMELESS, we examined the relationship between TIMELESS expression and the survival outcomes of patients with different malignancies. The TCGA analysis found that cancers of the ACC, LGG, LIHC, LUAD, MESO, SARC, and SKCM have a poor prognosis for overall survival (OS) when TIMELESS is highly expressed. The disease-free survival (DFS) analysis data for the TCGA cases of ACC, LGG, LIHC, and LUAD revealed a strong association between high TIMELESS expression with a poor survival prognosis (Fig. 3b).

### Genetic alteration analysis

**Fig. 4:**
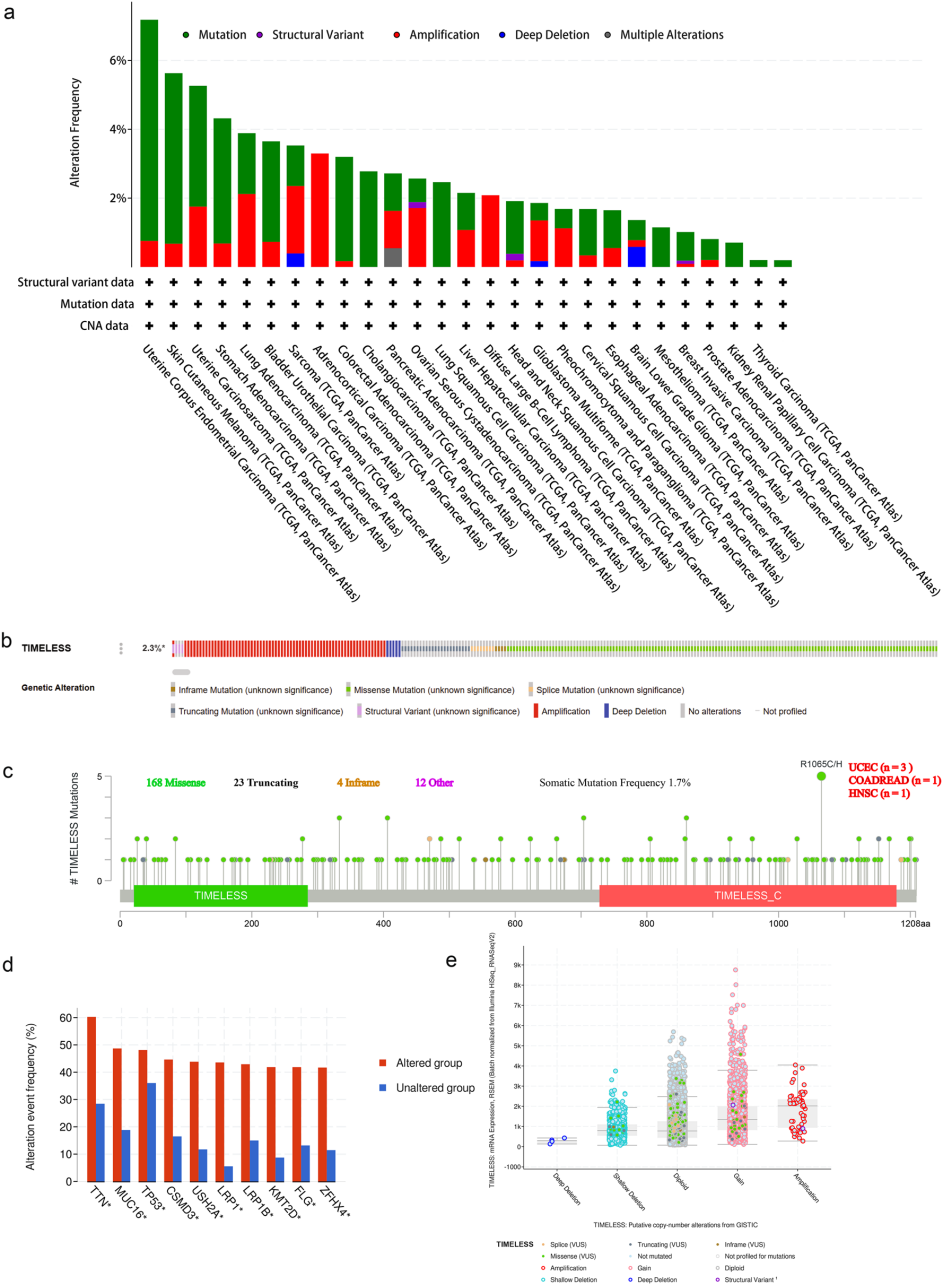

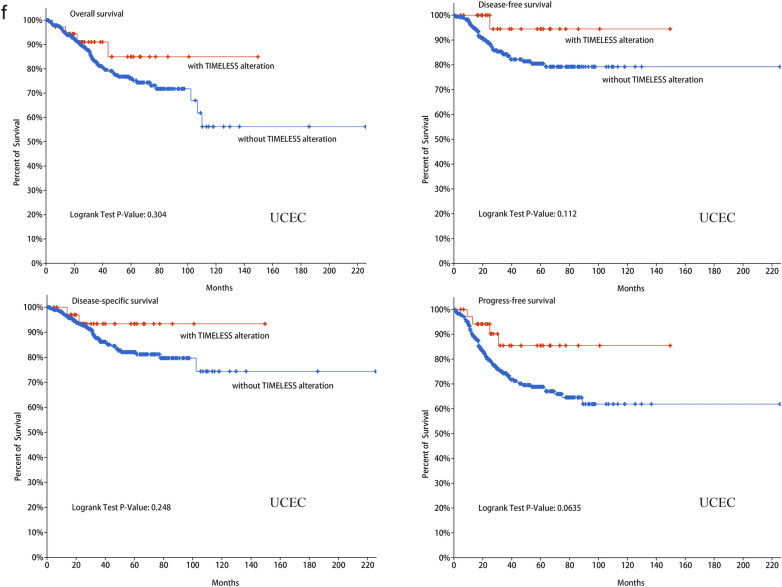
Genetic alteration characteristics of TIMELESS in different tumors of TCGA. **a** Alteration frequency of TIMELESS with different types of mutation in various cancer types. **b** Summary of different genetic alteration types of TIMELESS (Different colors refers to different types of TIMELESS genetic alterations). **c** Mutation types, sites and sample numbers of the TIMELESS genetic alterations. **d** Co-occurrence of genetic mutations in tumors with TIMELESS alterations (*Altered genes). **e** Correlated alteration types and putative copy-number alterations of TIMELESS in pan-cancer. **f** Potential correlation between mutation status of TIMELESS and overall, disease-specific, disease-free and progression-free survival of UCEC

In cBioPortal, the prevalence and types of mutations were studied. The most common TIMELESS alteration was seen in uterine corpus endometrial cancer (UCEC), with amplification accounting for the majority of cases, as illustrated in Fig. 4a. Of the four alteration types—mutation, structural variant, amplification, and deep deletion—mutation accounted for the majority of cases in UCEC patients. Amplification, miss mutation, and deep deletion were the three main genetic modification types of TIMELESS (Fig. 4b). In addition, the genetic alterations for TIMELESS were displayed in Fig. 4c together with their mutation sites, kinds, and sample numbers. Moreover, R1065C/H change was most frequently observed in UCEC, followed by COAD and HNSC, and the predominant form of alteration was the TIMELESS missense mutation. Genomic alternations co-occurrence analysis revealed that the TIMELESS-altered group had higher frequent changes of multiple genes, including TTN, MUC16, TP53, CSMD3, USH2A, LRP1, LRP1B, LMT2D, FLG, and ZFHX4 (Fig. 4d). Amplification, shallow deletion, and gain function were among the potential copy-number modifications of TIMELESS from GISTIC that may have altered gene expression (Fig. 4e). Figure 4f demonstrates that instances with TIMELESS change had a better prognosis for PFS (*P* = 0.0635) than cases with UCEC without the alteration, but not for disease-specific survival (DSS), (*P* = 0.248), DFS (*P* = 0.112) and OS (*P* = 0.304).

### Immune infiltration analysis

**Fig. 5:**
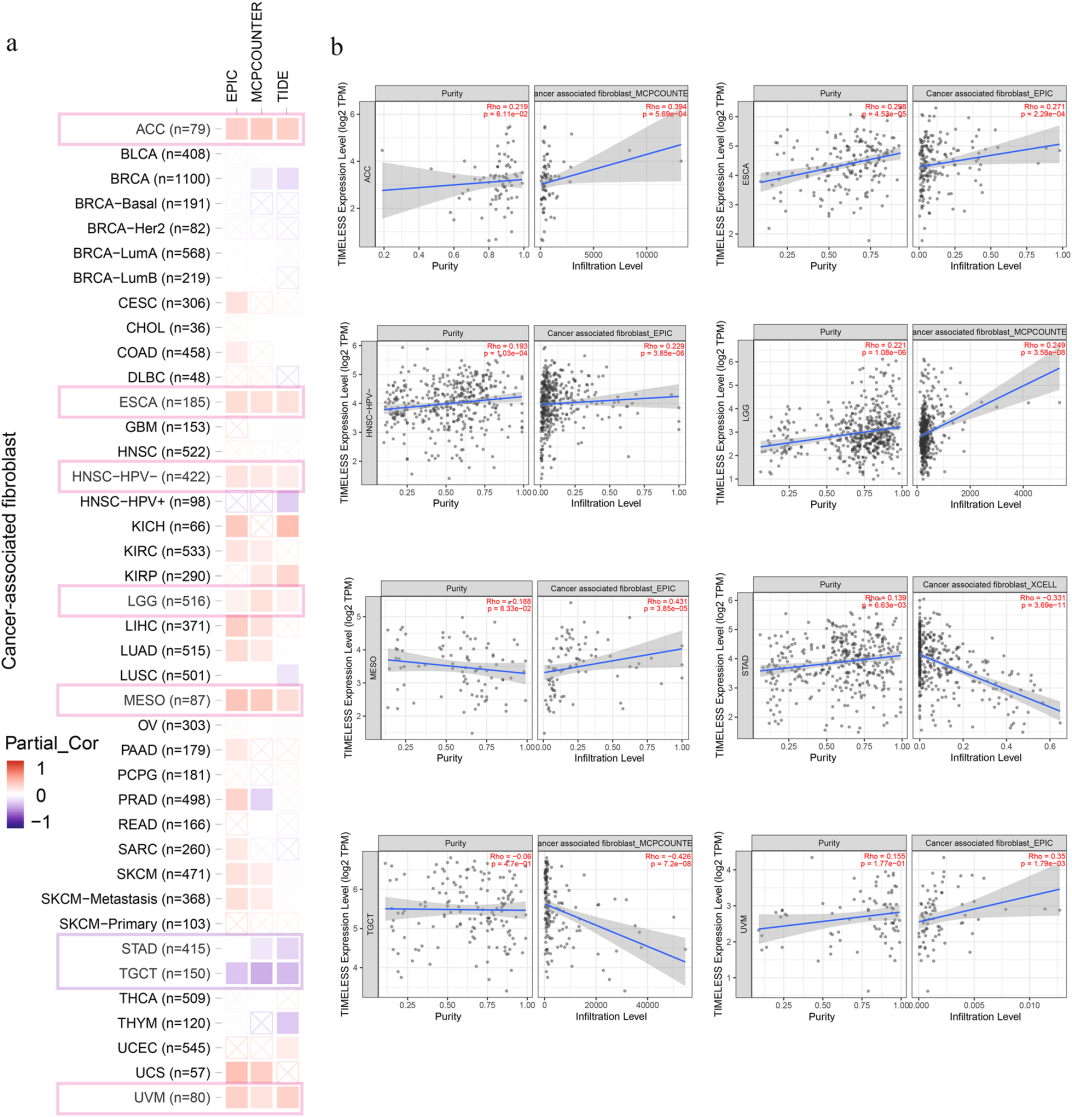
Correlation analysis between TIMELESS expression and immune infiltration of cancer-associated fibroblasts (CAF). Different algorithms were used to explore the potential correlation between the expression level of the TIMELESS gene and the infiltration level of CAF across all types of cancer in TCGA

The TIMER, CIBERSORT, MCPCOUNTER, and EPIC algorithms were employed in this study to examine any possible connections between the level of immune cell infiltration and the expression of the TIMELESS gene in various TCGA cancer types. For the TCGA tumors of ACC, ESCA, and HNSC [Human papillomavirus], LGG, MESO, and UVM, we discovered a statistically significant positive correlation between TIMELESS expression and the estimated infiltration value of CAFs, but we found a negative correlation for TGCT and STAD (Fig. 5a). Using the MCPCOUNTER algorithm, we discovered an intriguing correlation between the level of TIMELESS expression in LGG and the infiltration of cancer-associated fibroblasts (Fig. 5b, cor = 0.249, *P* = 3.58e−08).

**Fig. 6:**
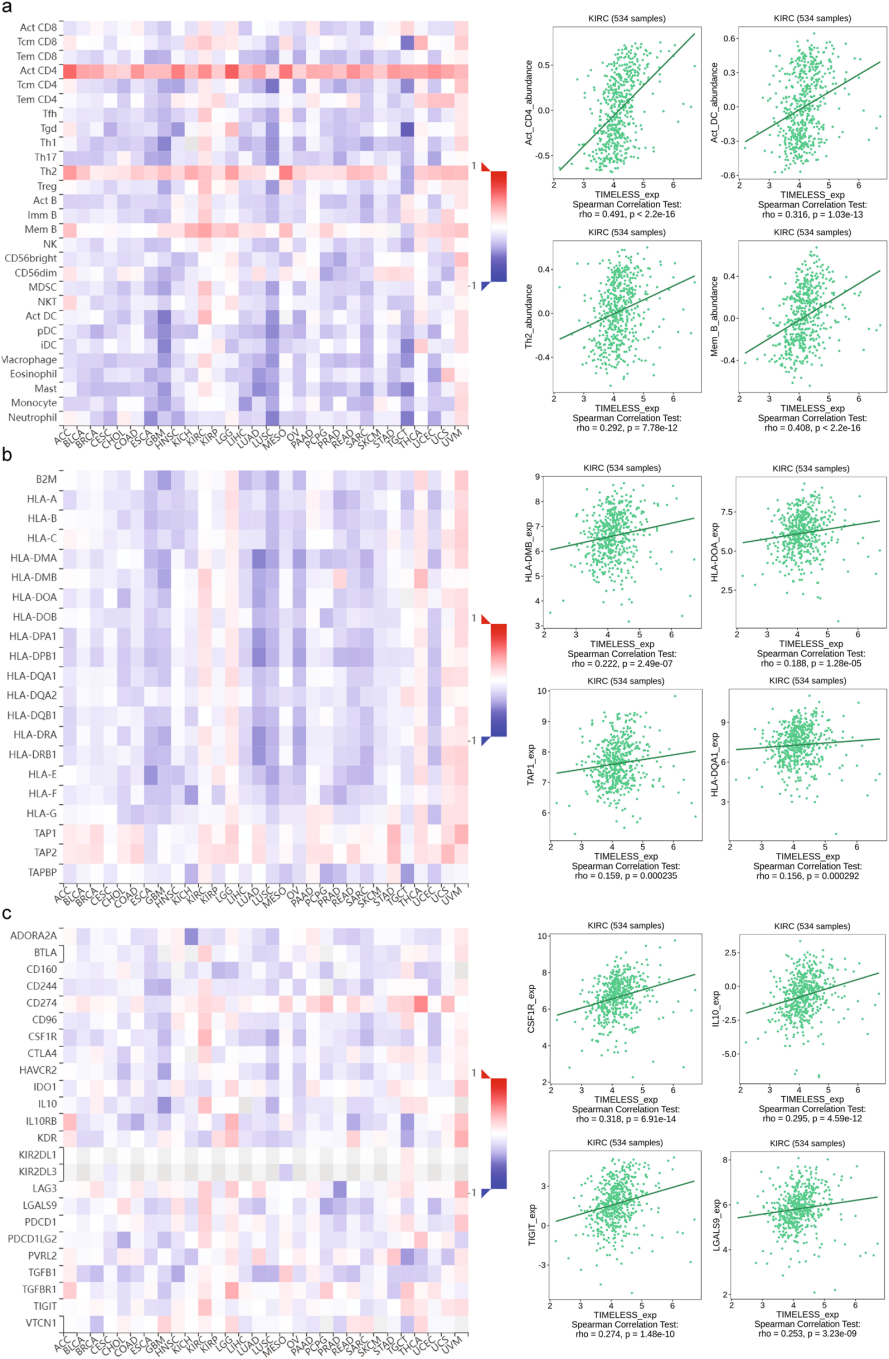

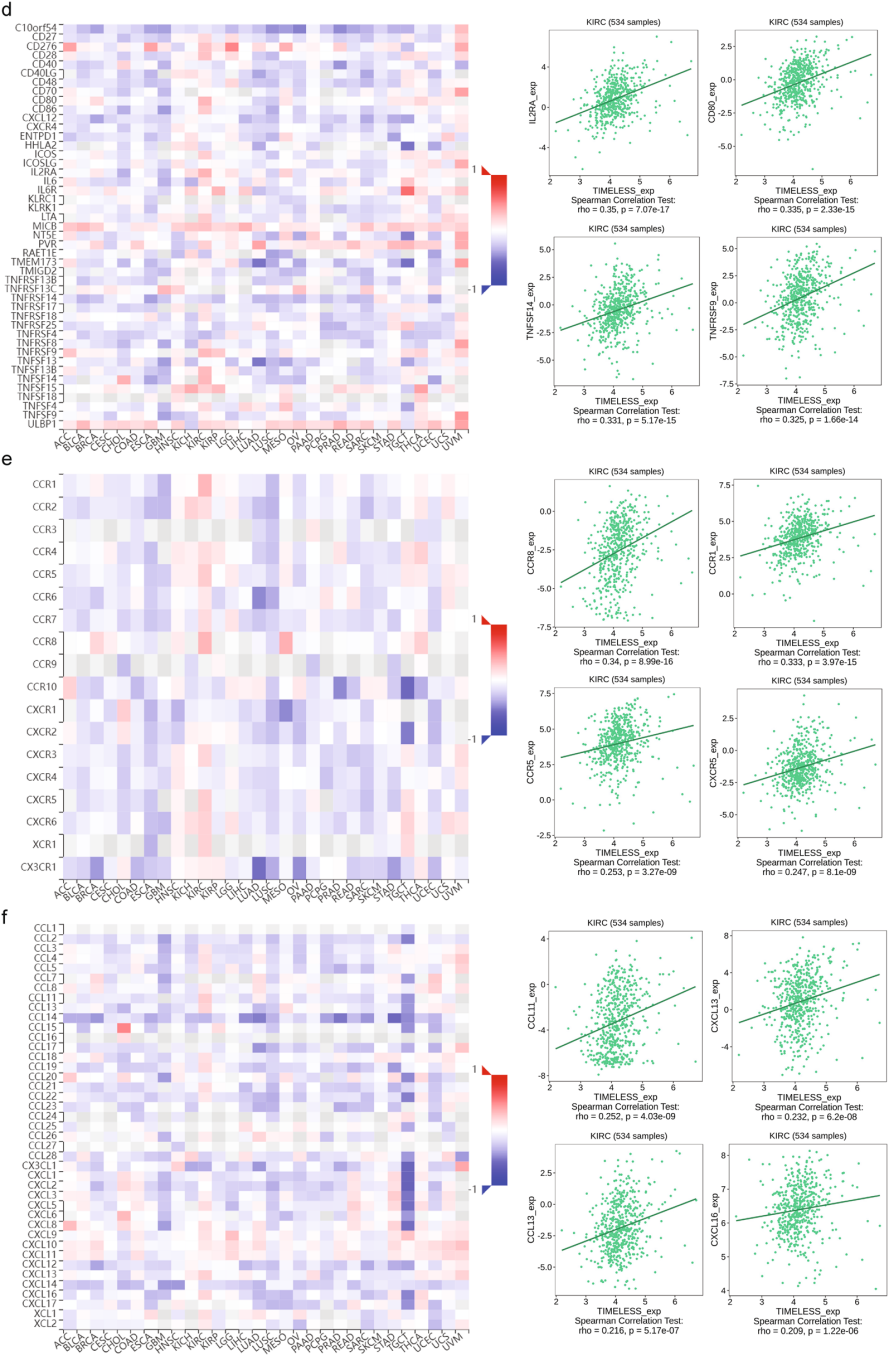
Correlations of TIMELESS expression and immune-infiltrating levels of 28 cell types (**a**), MHC molecules (**b**), immunoinhibitors (**c**), immunostimulators (**d**), receptors (**e**), and chemokines (**f**) were calculated by TISIDB

The associations between TIELESS expression and immune-infiltrating levels of 28 cell types or immune-related genes in 33 malignancies were further investigated using gene co-expression analysis. MHC molecules, immunoinhibitors, immunostimulators, chemokine receptor proteins, and chemokines were all encoded by the genes that were examined. As shown in Fig. 6a–f, the heatmap produced revealed that nearly all immune-related genes were co-expressed with TIMELESS in all types of tumors, especially in KIRC, and that the majority were strongly linked with TIMELESS (*P* < 0.05).

### Correlation of TIMELESS expression with TMB and MSI

**Fig. 7:**
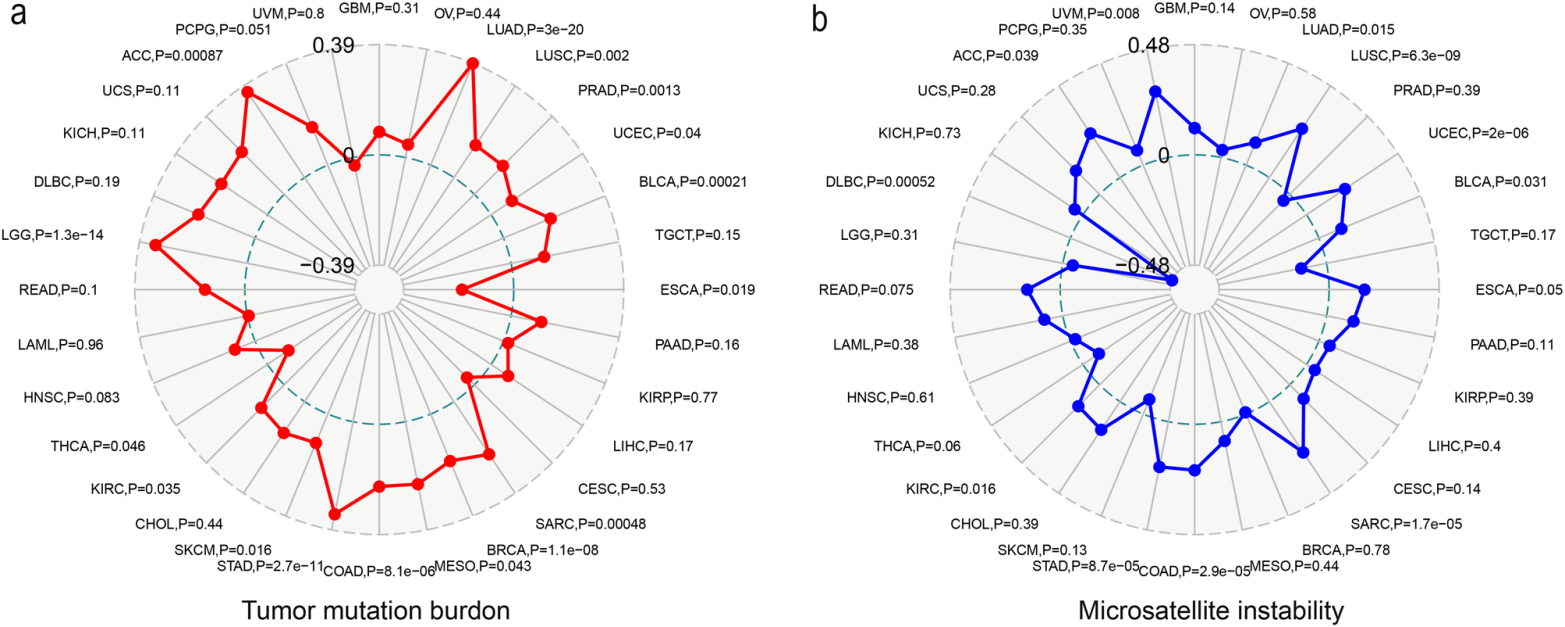
Correlation of TIMELESS expression with TMB level and MSI event. **a** Radar map of the relationship between TIMELESS expression and TMB levels. **b** Radar map of the relationship between TIMELESS expression and MSI event

The relationship between TIMELESS expression, TMB, and MSI-all of which have a strong correlation with the sensitivity to immune checkpoint inhibitors (ICIs) across malignancies—was then examined. The findings demonstrated that TMB expression and TIMELESS expression were related in many malignancies (*n* = 17, *P* < 0.05). Overall, TMB and TIMELESS expression were positively associated in fifteen different cancer types., including ACC, LUAD, LUSC, PRAD, UCEC, BLCA, SARC, BRCA, MESO, COAD, STAD, SKCM, KIRC, HNSC and LGG (Additional file 3: Table S2; Fig. 7a), and negatively correlated with TMB in ESCA and THCA, (Additional file 3: Table S2; Fig. 7a). We further found that TIMELESS expression was positively correlated with MSI in 12 cancer types, including ACC, UVM, LUAD, UCEC, BLCA, ESCA, SARC, COAD, STAD, KIRC, READ, and DLBC (Additional file 4: Table S3; Fig. 7b), and negatively correlated with MSI in LGG (Additional file 4: Table S3; Fig. 7b).

### Relationship between TIMELESS expression and drug sensitivity

**Fig. 8:**
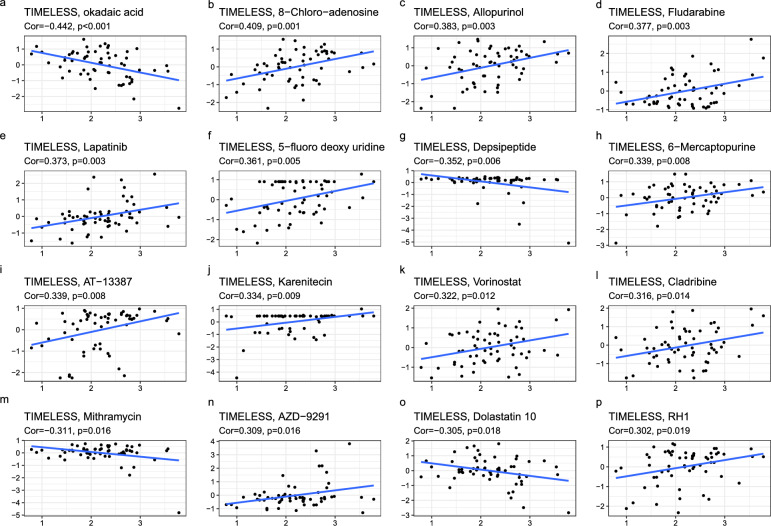
Relation of TIMELESS expression with drug sensitivity. The TIMELESS was linked to the sensitivity of (**a**) okadaic acid, (**b**) 8-chloro-adenosine, (**c**) allopurinol, (**d**) fludarabine, (**e**) lapatinib, (**f**) 5-fluoro deoxy uridine, (**g**) depsipeptide, (**h**) 6-mercaptopurine, (**i**) AT-13387, (**j**) karenitecin, (**k**) vorinostat, (**l**) cladribine, (**m**) mithramycin, (**n**) AZD-9291, (**o**) dolastatin 10, and (**p**) RH1

Investigations were also conducted into the possible link between TIMELESS expression and medication susceptibility in human malignancies (Fig. 8). The findings showed that TIMELESS was strongly associated with the sensitivity of numerous medications in a good way, including 8-chloro-adenosine (Fig. 8b), allopurinol (Fig. 8c), fludarabine (Fig. 8d), lapatinib (Fig. 8e), 5-fluoro deoxy uridine (Fig. 8f), 6-mercaptopurine (Fig. 8h), AT-13387 (Fig. 8i), karenitecin (Fig. 8j), vorinostat (Fig. 8k), cladribine (Fig. 8l), AZD-9291 (Fig. 8n), and RH1 (Fig. 8p), while was adversely correlated with the sensitivity to okadaic acid (Fig. 8a), depsipetide (Fig. 8g), mithramycin (Fig. 8m), and dolastatin (Fig. 8o). These findings suggested that TIMELESS would be a useful predictor of the sensitivity to many anti-cancer drugs, including allopurinol, fludarabine, lapatinib, and 6-mercaptopurine, which have all been used often in clinical cancer management.

### Functional enrichment analysis of TIMELESS-related and co-expression genes

**Fig. 9:**
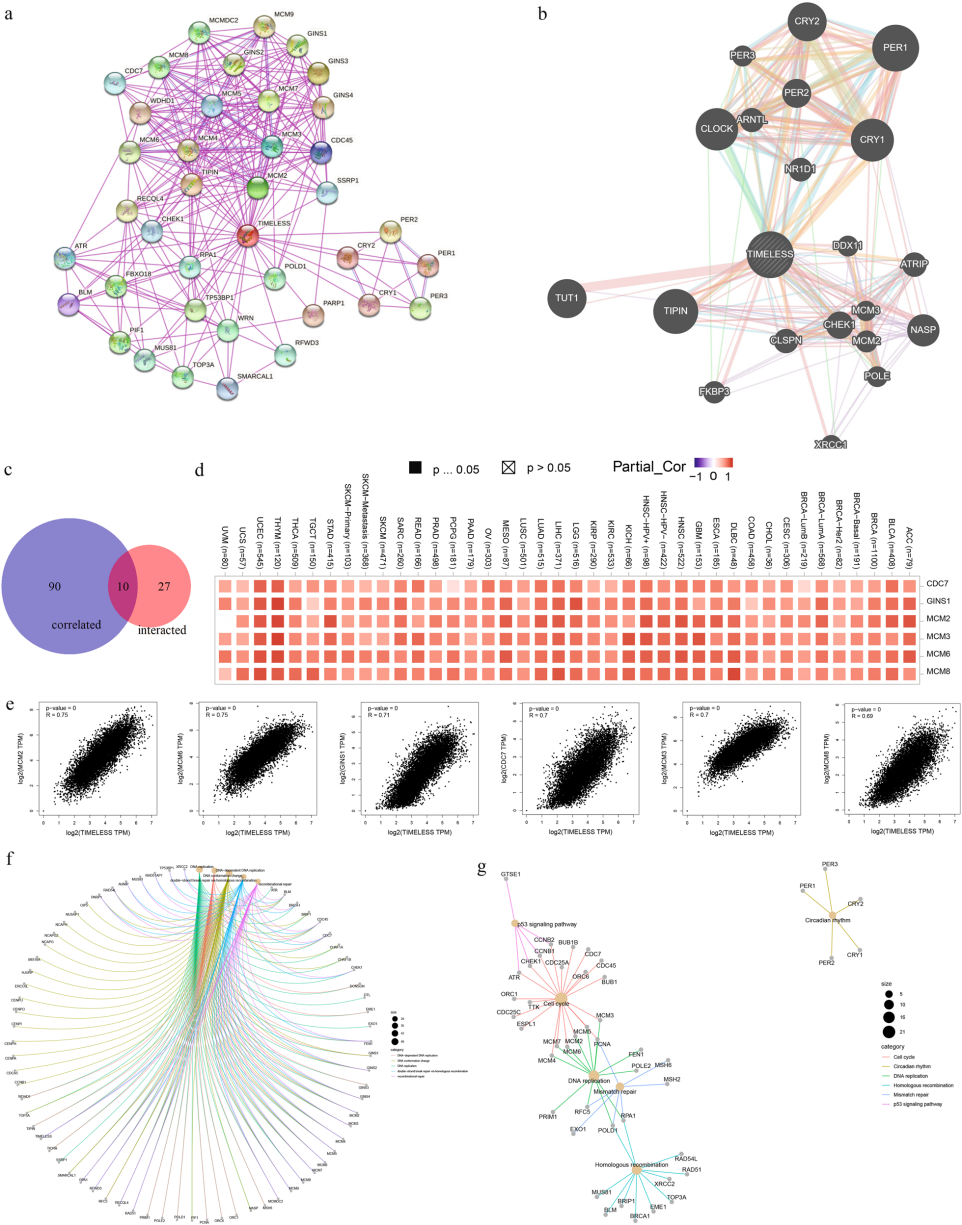
TIMELESS-related gene enrichment analysis. **a** We first obtained the available experimentally determined TIMELESS-binding proteins using the STRING tool. **b** Using the GeneMANIA to predict the functional network of TIMELESS. **c** Intersection analysis of the TIMELESS-binding and correlated genes was conducted. **d** The corresponding heatmap data in the detailed cancer types are displayed between TIMELESS and selected targeting genes, including CDC7, MCM2, MCM3, MCM6, MCM8, and GINS1. **e** Using the GEPIA2 approach, we also obtained the top 100 TIMELESS-correlated genes in TCGA projects and analyzed the expression correlation between TIMELESS and selected targeting genes, including MCM2, MCM6, GINS1, CDC7, MCM3 and MCM8. **f** Based on the TIMELESS-binding and interacted genes, The cnetplot for the biological process data in GO analysis is shown. **g** KEGG pathway analysis was also performed

To better understand the molecular mechanism of the TIMELESS gene in carcinogenesis, we tried to filter out the targeting TIMELESS-binding proteins and the TIMELESS expression-correlated genes for functional enrichment analyses. Firstly, we obtained a total of 37 TIMELESS-binding proteins through the STRING tool, which was supported by experimental evidence and median confidence score (0.400). The protein interaction networks is depicted, Fig. 9a. GeneMANIA database was utilized to predict the function network of TIMELESS (Fig. 9b). The top 100 genes that linked with TIMELESS expression were then obtained by combining all of the TCGA's tumor expression data using the GEPIA2 program. we utilized the GEPIA2 tool to combine all tumor expression data of TCGA and obtained the top 100 genes that correlated with TIMELESS expression (Additional file 5: Table S4). Ten genes shared by the two groups mentioned above were discovered by an intersection analysis (Fig. 9c), namely CDC45, CDC7, CHEK1, GINS1, MCM2, MCM3, MCM5, MCM6, and PIF1. The heatmap revealed a positive correlation between the TIMELESS expression levels of MCM2, MCM6, GINS1, CDC7, MCM3, and MCM8 (Fig. 9d). As shown in Fig. 9e, the TIMELESS expression level was positively correlated with that of MCM2 (Minichromosome Maintenance Complex Component 2) (*R* = 0.75), MCM6 (*R* = 0.75), GINS1 (GINS Complex Subunit 1) (*R* = 0.71), CDC7 (Cell Division Cycle 7) (*R* = 0.7), MCM3 (*R* = 0.7) and MCM8 (*R* = 0.69) genes (all *P* value = 0). In the majority of the specific cancer types, the associated heatmap data likewise demonstrated a positive association between TIMELESS and the aforementioned six genes.

In order to perform GO and KEGG enrichment analysis, the two data sets were also joined. Most of these genes, according to the GO enrichment analysis, are connected to biological pathways or processes involved in RNA metabolism, including DNA replication, DNA conformation change, recombinational repair, and others (Additional file 6: Table S5, Fig. 9f). The KEGG further indicated that “cell cycle” and “DNA replication”, “circadian rhythm”, “mismatch repair” and “homologous recombination” might be involved in the effect of TIMELESS on carcinogenesis (Additional file 6: Table S5, Fig. 9g).

### Experimental validation using IHC staining

**Fig. 10:**
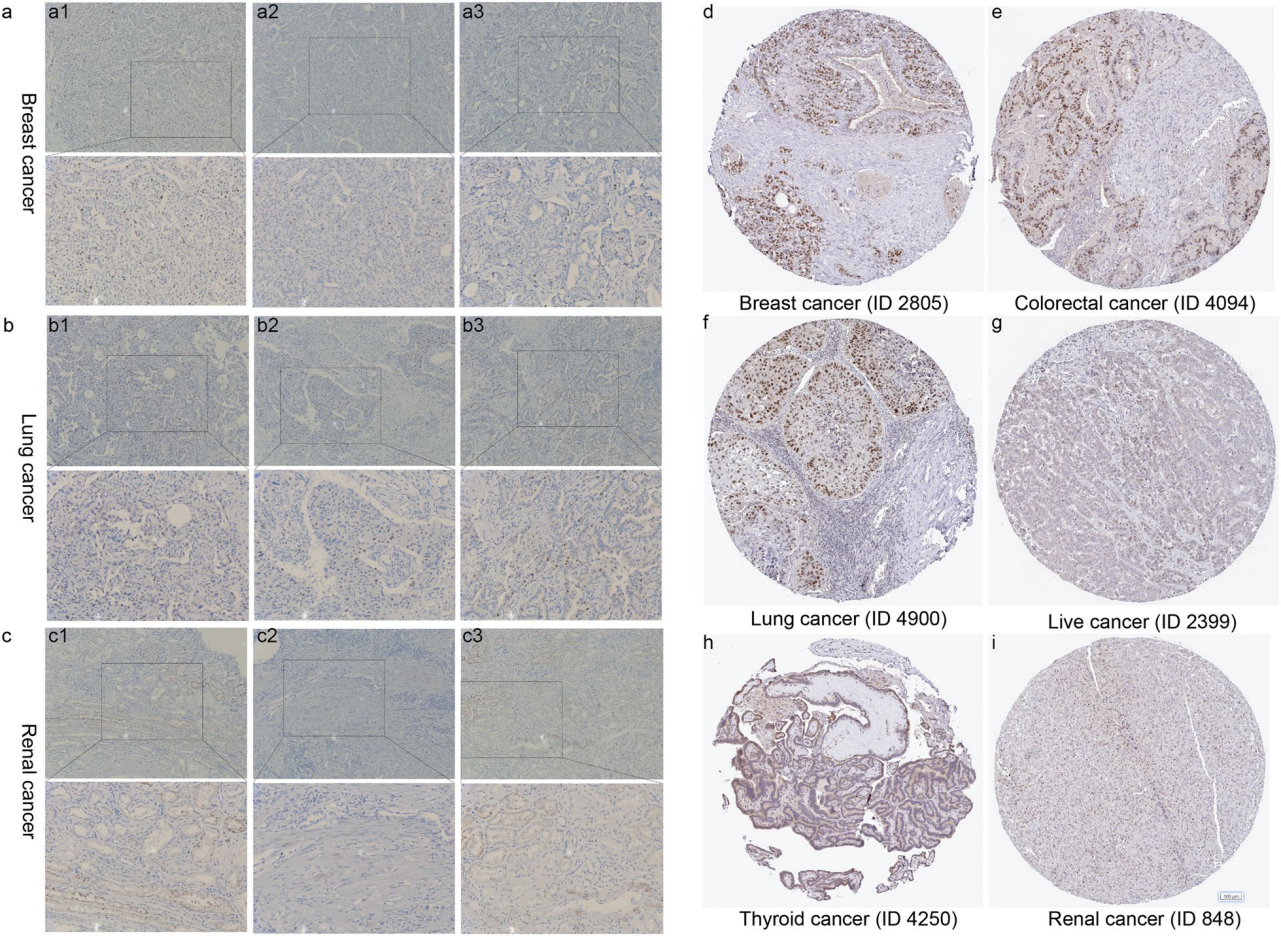
IHC staining. Representative IHC images of TIMELESS expression in human cancers. **a**–**c** Clinical specimens (100× and 200×): breast cancer (**a**), lung cancer (**b**) and renal cancer (**c**). **d**–**i** HPA database (100 nm): breast cancer (**d**), colorectal cancer (**e**), lung cancer (**f**), liver cancer (**g**), thyroid cancer (**h**), and renal cancer (**i**)

We also carried out immunohistochemical labeling of the TIMELESS in numerous clinical tumor tissues and the HPA database to confirm the preceding conclusion that aberrant TIMELESS expression was substantially connected with poor prognosis in certain cancers. IHC testing verified that the TIMELESS expression of the TIMELESS protein was moderately positive in breast cancer, lung cancer, and renal cancer (Fig. 10a–c) from clinical cancer specimens. In addition, HPA database demonstrates that TIMELESS protein was moderate to strong positively in breast cancer, colorectal cancer, lung cancer, liver cancer, renal cancer, and thyroid cancer (Fig. 10d–i).

## Discussion

Large-scale and multi-omics cancer data sets, such as TCGA, UCSC, and GEO, which contain functional genomics data sets of various types of tumors, have given researchers a platform for pan-cancer study. The pan-cancer analysis is essential and useful for contrasting the similarities and differences among various malignancies, which in turn serves to throw fresh light on our understanding of how cancer develops and to offer a novel understanding of tumor biomarkers. Numerous research has shed fresh light on the human pan-cancer whole-genome analysis and showed a direct connection between carcinogenesis and mutations and copy number changes, which is crucial for the detection and management of different malignancies [27–29]. The TIMELESS gene was first discovered as a molecule in the Drosophila melanogaster circadian clock through forward genetic screening by Leslie B. Vosshall in 1994 [30]. Due to its similar sequence, mammalian TIMELESS was identified as an essential circadian clock protein in flies. As an evolutionarily conserved core circadian clock gene in primates, TIMELESS plays multifaced roles in physiological and pathological processes, including embryonic development, DNA damage response, and DNA replication. In addition to its role in circadian rhythmicity, the TIMELESS protein also plays a role in DNA replication, the DNA damage response (DDR), embryonic development, cell cycle progression, as well as the maintenance of telomere length and integrity.

The circadian clock is a molecular time-keeping system that has survived evolution and controls daily oscillations of biological processes and behaviors, including sleep and hormone secretion [13]. Circadian disturbance has a deleterious impact on physiology and poses a threat to world health, manifesting among other things in proliferative, metabolic, and immunological illnesses [31, 32]. The period (PER) gene and its protein participate in a transcription–translation negative feedback loop that causes the TIMELESS mRNA and protein to oscillate regularly with time. The products of the important clock genes CLOCK, BMAL, PER1, PER2, and PER3 interact with the TIMELESS protein as well [33]. Human TIMELESS protein interacts with the cell cycle checkpoint proteins CHK1, ATR, and the ATR-interacting protein (ATRIP), as well as the circadian proteins CRY2/1 and PER1/2/3 [8]. In addition, exposure to DNA-damaging substances including hydroxyurea (HU) and UV radiation enhanced the interaction of TIMELESS with checkpoint proteins. The G2/M and intra-S cell cycle checkpoints depend heavily on TIMELESS protein. TIMELESS protein binds to the ATRIP component on ATR, a protein kinase sensitive to DNA damage, concerning the G2/M checkpoint. Moreover, Barrio-Alonso et al. [34] found that the expression of the circadian protein TIMELESS exhibits circadian rhythmicity in the mammalian hippocampus. TIMELESS acts as a chromatin-bound protein that targets synaptic-plasticity-related genes, such as phosphodiesterase 4B (Pde4b) and promotes Pde4b transcription, and negatively regulates cAMP signaling, thereby regulating the function of the AMPA receptor GluA1 function and affecting synaptic plasticity. Consequently, TIMELESS may be crucial to the development and progression of cancer.

Our study found that TIMELESS was aberrantly expressed and related to poorer survival in various malignant tumors, such as ACC, LGG, LIHC, and SARC. Liu Sai-Nan et al. [21] discovered that TIMELESS was overexpressed and associated with poorer survival in NPC, as well as conferred resistance to cisplatin-induced apoptosis and promoted the epithelial–mesenchymal transition (EMT), which could be a valuable prognostic factor and therapeutic target in NPC. According to a study on cervical cancer, TIMELESS overexpression is associated with lymphovascular space involvement, pelvic lymph node metastases, and worse OS and DFS. This suggests that TIMELESS may be a possible prognostic biomarker for cervical cancer patients [35]. Overexpression of TIMELESS was substantially linked to a worse prognosis and a more advanced tumor stage in breast cancer [36].

Studies in genetic mice models and cell lines have outlined the interactions between the circadian clock and numerous pathways connected to oncogenes and tumor suppressors, such as c-Myc, Ras, PTEN, and p53. The circadian clock predominates metabolic signal pathways regulating—oxidation, lipogenesis, amino acid absorption, glucose consumption, and glucose utilization. Deregulation of circadian rhythms thus impacts cancer metabolism and consequent cell proliferation, opening up new therapeutic possibilities [11]. The TIMELESS mutation caused arrhythmic behavior, namely lack of ability to establish proper circadian rhythms. In this study, there are many genetic alterations of TIMELESS, including amplification, miss mutation, and deep deletion, which may bring on a worse survival prognosis.

It has been suggested that CAF have an impact on how various immune cells interact with malignancies [37, 38]. Studies have shown that circadian rhythm significantly affects immune responses [39]. Likewise, our study showed that TIMELESS was significantly associated with the CAF in various cancer, including ACC, ESCA, HNSC, LGG, MESO, UVM, STAD, and TGCT. A study performed by Xing et al. [40] showed that high expression of TIMELESS was associated with immune cells especially macrophage infiltration in ovarian cancer. In addition, TIMELESS and its constructive binding protein TIPIN (TIMELESS-interacting protein) are critical for early embryonic development. Studies showed that TIMELESS is expressed in embryonic tissues, and involved in murine lung, kidney, and urethral bud branching morphogenesis and epithelial organogenesis [41–43]. In addition, TIMELESS is crucial for DNA replication. TIMELESS shares structural and functional similarities with a family of proteins that are crucial for DNA synthesis, S-phase-dependent checkpoint activation, and chromosomal cohesion in eukaryotes. At the level of the DNA replication fork complex, these processed are coordinated [9, 44]. Precatenation and fork rotation help unwind at hard-replicate regions, but they also fundamentally interfere with proper chromosomal duplication, and TIMELESS–TIPIN inhibits these processes [45].

According to earlier research, chemotherapy and TIMELESS are highly connected in several types of human cancer [46, 47]. TIMELESS is significantly connected with the susceptibility of numerous anti-cancer targeted treatment medications, including fludarabine, lapatinib, and 6-mercaptopurine, as this study’s findings have shown. These results suggest that in some human malignancies, TIMELESS exhibits intriguing promise as a novel therapeutic target and a predictive biomarker for anti-cancer therapy sensitivity. The probable process by which TIMELESS modulated medication sensitivity, however, is still unclear, suggesting a potential future area of research for TIMELESS. Furthermore, Shen et al. [48] found that TIMELESS was down-regulated in cellular senescence, and deletion of clock protein TIMELESS may promote the cellular senescence and exacerbate genome instability at the onset of senescence, suggesting that TIMELESS inhibitors have potential applications in cancer prevention. Moreover, there is still no relevant evidence on the relationship between TIMELESS expression and the effectiveness of immunotherapy. We, therefore, deduced that TIMELESS may take important roles in cancer immunotherapies and alter the efficacy of immunotherapeutics. Higher levels of TMB and MSI are typically regarded as significant biomarkers correlated with the high therapeutic efficacy in numerous immune checkpoint inhibitors [25, 26]. Our research provided evidence for a potential association between TIMELESS expression and MSI and TMB in several malignancies. According to our research, TMB and MSI were substantially correlated with TIMELESS expression in COAD and lung cancer, indicating that patients who express TIMELESS substantially may respond better to immune checkpoint inhibitors.

Nevertheless, this excellent study has several shortcomings. First of all, the data enrolled in our study was primarily gathered from open-access databases and clinical samples of several cancer kinds, but it has to be confirmed with further clinical patients and contrasted with corresponding normal tissues. In addition, it is yet unclear how TIMELESS affects the tumor immune microenvironment and what functions it plays in a particular form of cancer. Future research is required to confirm these findings from in vitro and in vivo trials.

Taken together, the findings of our study showed statistically significant associations between TIMELESS expression and clinical prognosis, immune cell infiltration, tumor mutational burden or microsatellite instability, and drug sensitivity across a range of tumors, which helps to explain the function of TIMELESS in tumorigenesis and immunotherapy from the viewpoint of clinical tumor samples. As a result, TIMELESS may be crucial as a new and potent immunological and prognostic biomarker across several malignancies.

### Supplementary Information

**Additional file 1: Fig. S1.** TIMELESS expression levels in human cancers. (a–d) TIMELESS RNA expression levels in different tissues in consensus data set, HPA data set, GTEx data set and FANTOM5 data set analyzed by the TIMER2.0 database, respectively. (e) Based on the CPTAC database, the expression level of TIMELESS phosphoprotein between normal tissue and primary tissue of clear cell RCC, glioblastoma multiforme, head and neck squamous cell carcinoma and hepatocellular carcinoma were also analyzed.

**Additional file 2: Table S1.** TIMELESS gene expression in 33 types of cancer and normal controls.

**Additional file 3: Table S2.** Correlation of TIMELESS expression with TMB.

**Additional file 4: Table S3.** Correlation of TIMELESS expression with MSI.

**Additional file 5: Table S4.** Top100 correlated genes with TIMELESS from GEPIA2.

**Additional file 6: Table S5.** GO and KEGG analysis.

## Data Availability

The data involved in this study and supplementary materials are openly available in the following databases and the corresponding websites: HPA: https://www.proteinatlas.org/; TIMER2.0: http://timer.cistrome.org/; GEPIA2: http://gepia2.cancer-pku.cn/#analysis; UALCAN: http://ualcan.path.uab.edu/analysis-prot.html; BioGPS: http://biogps.org; cBioPortal: https://www.cbioportal.org/; TISIDB: http://cis.hku.hk/TISIDB/index.php; Sangerbox: http://past20.sangerbox.com; CallMiner™ database: https://discover.nci.nih.gov/cellminer/home.do; STRING database: https://string-db.org. For further information, please contact the authors.

## Abbreviations

ACC: Adrenocortical carcinoma
BLCA: Bladder urothelial carcinoma
BRCA: Breast carcinoma
CESC: Cervical and endocervical cancers
CHOL: Cholangiocarcinoma
COAD: Colon adenocarcinoma
DLBC: Diffuse large B-cell lymphoma
ESCA: Esophageal carcinoma
GBM: Glioblastoma multiforme
HNSC: Head and neck squamous cell carcinoma
NPC: Nasopharyngeal carcinoma
LGG: Low-grade glioma
KICH: Kidney chromophobe
KIRC: Kidney renal clear cell carcinoma
KIRP: Kidney renal papillary cell carcinoma
LAML: Acute myeloid leukemia
LIHC: Liver hepatocellular carcinoma
LUAD: Lung adenocarcinoma
LUSC: Lung squamous cell carcinoma
MESO: Mesothelioma
OV: Ovarian serous cystadenocarcinoma
PAAD: Pancreatic adenocarcinoma
PCPG: Pheochromocytoma and paraganglioma
PRAD: Prostate adenocarcinoma
SARC: Sarcoma
SKCM: Skin cutaneous melanoma
STAD: Stomach adenocarcinoma
TGCT: Testicular germ cell tumors
THCA: Thyroid carcinoma
THYM: Thymoma
UCEC: Uterine corpus endometrioid carcinoma
UCS: Uterine carcinosarcoma
UVM: Uveal melanoma
HPV: Human papillomavirus
CAF: Cancer-associated fibroblasts
TMB: Tumor mutation burden
MSI: Microsatellite instability
IHC: Immunohistochemistry
MMR: Mismatch repair
OS: Overall survival
DFS: Disease-free survival
PFS: Progression-free survival
DSS: Disease-specific survival
CAN: Copy number alteration
GO: Gene ontology
KEGG: Kyoto Encyclopedia of Genes and Genomes
PPI: Protein–protein interaction
EMT: Epithelial–mesenchymal transition
HPA: Human Protein Atlas
TIMER: Tumor Immune Estimation Resource
GEPIA: Gene Expression Profiling Interactive Analysis
GTEx: Genotype-Tissue Expression
TCGA: The Cancer Genome Atlas
ICGC: International Cancer Genome Consortium
CPTAC: Clinical Proteomic Tumor Analysis Consortium
TISIDB: Tumor-immune system interactions database
