# Supplementary figures and images for: An integrative evaluation of circadian gene TIMELESS as a pan-cancer immunological and predictive biomarker
Source: Eur J Med Res. 2023 Dec 5;28:563. doi: 10.1186/s40001-023-01519-3 (PMC10696727; doi:10.1186/s40001-023-01519-3)

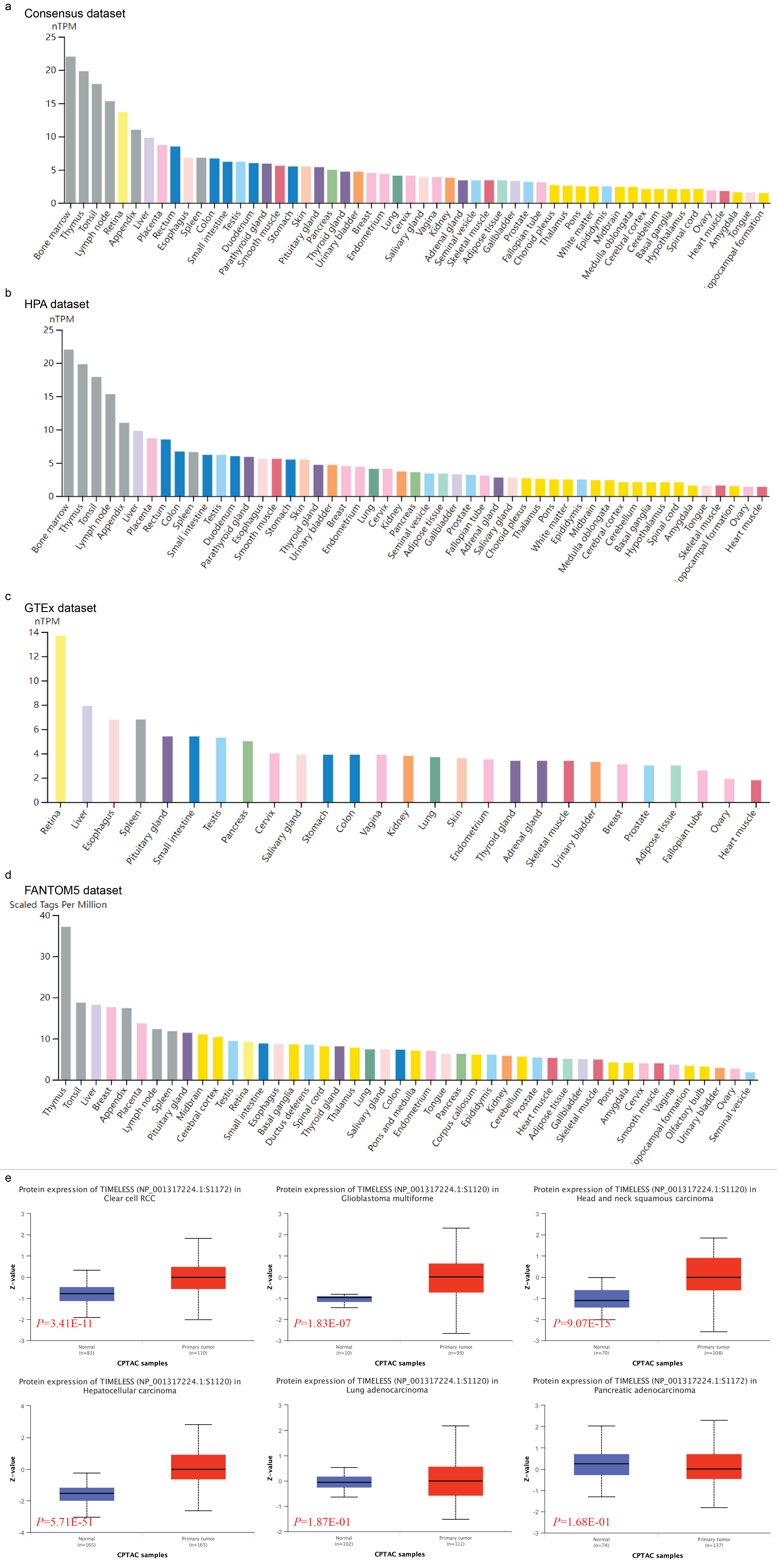

Supplement: Supplementary file 1 — Additional file 1: Fig. S1. TIMELESS expression levels in human cancers. (a–d) TIMELESS RNA expression levels in different tissues in consensus data set, HPA data set, GTEx data set and FANTOM5 data set analyzed by the TIMER2.0 database, respectively. (e) Based on the CPTAC database, the expression level of TIMELESS phosphoprotein between normal tissue and primary tissue of clear cell RCC, glioblastoma multiforme, head and neck squamous cell carcinoma and hepatocellular carcinoma were also analyzed. [file 40001_2023_1519_MOESM1_ESM.png]
